# Expression of Bioactive Lunasin Peptide in Transgenic Rice Grains for the Application in Functional Food

**DOI:** 10.3390/molecules23092373

**Published:** 2018-09-17

**Authors:** Guixing Ren, Yuqiong Hao, Yingying Zhu, Zhenxing Shi, Gang Zhao

**Affiliations:** 1 College of Pharmacy and Biological Engineering, Chengdu University, No. 1 Shilling Road, Chenglo Avenue, Longquan District, Chengdu 610106, China; 2 Institute of Crop Science, Chinese Academy of Agricultural Sciences, No. 80 South Xueyuan Road, Haidian, Beijing 100081, China; haoyuqiong334@163.com (Y.H.); zhuying881020@163.com (Y.Z.); shizhenxing@caas.cn (Z.S.); 3 Precision Livestock and Nutrition Unit, Gembloux Agro-Bio Tech, TERRA Teaching and Research Centre, University of Liège, Passage des Déportés, 5030 Gembloux, Belgium; 4 Laboratory of Biomass and Green Technologies, Gembloux Agro-Bio Tech, University of Liège, Passage des Déportés, 5030 Gembloux, Belgium; 5 Key Laboratory of Coarse Cereal Processing, Ministry of Agriculture, Chengdu University, No.1 Shilling Road, Chenglo Avenue, Longquan District, Chengdu 610106, China

**Keywords:** lunasin, transgenic rice, UPLC-MS/MS, antioxidant activity, anti-inflammatory activity

## Abstract

Lunasin, a bioactive peptide initially isolated from soybean, has anticancer, anti-inflammatory, and antioxidant activity. Due its great application value, lunasin seems to be a candidate gene in improving the nutritional value of crops. In this study, lunasin was inserted into the rice genome to evaluate whether it was feasible to express lunasin using the rice expression system and improve the bioactivity of protein in rice for our needs. We generatedlunasin-overexpressing rice lines, and chose three independent transgenic rice lines for further study. The lunasin content in trans-lunasin rice detected by UPLC-MS/MS was 1.01 × 10^−3^ g·kg^−1^ dry rice flour with grease removal in the lunasin extracts. The antioxidant efficacy of LET (lunasin-enriched fraction from trans-lunasin rice) and PEW (peptide-enriched fraction from wild type rice) was compared. Due to the presence of lunasin, LET showed higher (*p* < 0.05) antioxidant activity than PEW. LET exhibited high DPPH radical scavenging activity (IC_50_ value, 8 g·L^−1^), strong ABTS^+^ radical scavenging activity (IC_50_ value, 1.18 g·L^−1^), and great oxygen radical scavenging activity (170 μmol·L^−1^ Trolox equivalents when the concentration reached 4 g·L^−1^). Moreover, LET presented significantly higher (*p* < 0.05) anti-inflammatory activity on macrophage cells, and the NO production and the release of pro-inflammatory cytokines (IL-6, MCP1, and TNF-α) were significantly inhibited by LET. However, because of the low purity, LET showed weaker antioxidant and anti-inflammatory activity when compared to the Lunasin standard. These results suggested that it is feasible to use the rice expression system to express the exogenous lunasin in rice, and lunasin-overexpressing rice seems to be a candidate resource for application in functional food. Rice rich in lunasin is beneficial for human health, and could be used as a functional food in the diets of cancer and obese patients in the future.

## 1. Practical Application

Trans-lunasin rice peptide extracts exhibited enhanced antioxidant activity and anti-inflammatory activity. The trans-lunasin rice seems to be a candidate resource for application in functional food.

## 2. Introduction

Lunasin, a 43-amino acid peptide with a molecular mass of 5.5 KDa, was initially isolated from soybean [1], and contains an Arg-Gly-Asp cell adhesion motif [2] as well as a conserved chromatin-binding region for binding to histone H3/H4 [3]. Lunasin presents nine Asps at its C-terminus end, the poly-Asp may be involved in antimitotic functions [3,4]. Studies have found that the lunasin peptide has anticancer, anti-inflammatory, antioxidant, and cholesterol-lowering activity. The anticancer activity may be due to the inhibition of the acetylation of histone H3 and H4 [3,5]. Anti-inflammatory activity is the reason for the release of pro-inflammatory cytokines in the NF-kB pathway being suppressed by lunasin [6]. Antioxidant activity is due to its abilities to scavenge the reactive oxygen species and inhibit linoleic acid oxidation [7]. Moreover, some studies have shown that the lunasin peptide has potential applications to lower the cholesterol and low-density lipoprotein levels through inhibiting the gene expression of some related proteins such as 3-hydroxy-3-methyl-glutaryl-CoA reductase, which plays an important role in cholesterol biosynthesis, and the LDL receptor, which functions in clearing the LDL cholesterol [8,9]. It has been demonstrated that the lunasin peptide not only exists in soybean, but also exists in other cereals such as quinoa [10], wheat [11], barley [12], and rye [13]. The identification of lunasin can be realized by UPLC-MS/MS, ELISA, Western blot, or other technical means. Through the UPLC-MS/MS technique, Nakurte first found that oat contained lunasin [14]. Using the same method, Rosa et al. found the existence of lunasin in amaranth mature grain, but the lunasin content was extremely low, only 4.8 to 7.2 μg/g dry powder [15].

In recent years, substances from soybean have been found to play an important role in the antitumor effect [16,17]. Dia VP purified lunasin from defatted soybean, and found that lunasin showed high anti-inflammation and antioxidant activity in the LPS-induced inflammations and inhibited the macrophages producing cytotoxic inflammatory mediators [18]. Galvez showed that serum LDL-C level of obese pig (LDL receptors defect type) could significantly decrease with the daily intake of 250 mg or 500 mg of lunasin extracts (the content of lunasin was above 20%). Due to the potential applications of lunasin, more and more scientists have tried to express it in other species. To date, many expression systems have been developed to express valuable recombinant proteins for industrial applications or academic research [19]. A previous study had attempted to express the lunasin peptide in *Escherichia coli* [20]. Keith R. Davis established a tobacco transient expression system which could produce GFP-lunasin at levels >100 mg·kg^−1^ fresh weight tissue, and the expressed recombinant lunasin showed enhanced anticancer activity [21]. Galvez et al. transferred the lunasin gene into pichia yeast for secretory expression, and obtained the high purity lunasin through SEC-IEC and affinity chromatography [8]. Ren et al. improved the expression of lunasin in pichia yeast by optimizing the fermentation process of pichia yeast, and obtained the recombinant lunasin peptide with a purity of 93% [22].

Rice, as a global crop, is generally regarded as safe for consumption. Expression of some exogenous genes in rice is feasible for our needs [23,24,25]. Nandi et al. provided a convenient and high-efficiency system to express human lactoferrin in transgenic rice for application in infant formula [26]. In this study, we introduced the lunasin gene into the rice genome and generated lunasin-overexpressing rice lines. The trans-lunasin peptide extracts exhibited enhanced antioxidant activity and anti-inflammatory activity. The lunasin gene seems to be a candidate gene in improving the nutritional value of rice for our use.

## 3. Materials and Methods

### 3.1. Reagent

The primers were synthesized by the Beijing Genomics Institute (Beijing, China). The lunasin standard was synthesized by the American Peptide Company (Sunnyvale, CA, USA). The primary rabbit polyclonal antibody lunasin epitope—EKHIMEKIQGRGDDDDD—was synthesized by the Sangon Biotech Corporation (Shanghai, China). Cetyl trimethyl ammonium bromide (CTAB), 2×Taq PCR MasterMix were purchased from GenStar BioSolutions Corporation (Beijing, China). Protease inhibitor cocktail, 1,1-diphenyl-2-picrylhydrazyl radical (DPPH), 2,2′-azino-bis (3-ethylbenzothiazoline-6-sulfonicacid) diammonium salt (ABTS), 6-hydroxy-2,5,7,8-tetramethylchromane-2-carboxylic acid (Trolox), and lipopolysaccharide (LPS) were purchased from the American Sigma-Aldrich Company (St. Louis, MO, USA). Goat anti-rabbit IgG-HRP was purchased from the American Thermo Fisher Scientific Corporation (Waltham, MA, USA). Mouse monocyte chemoattractant protein chemokine (C-C motif) ligand 2 (MCP1/CCL2) Simple Step Elisa Kit, mouse tumor cell necrosis factor-α (TNF-α), SimpleStep Elisa Kit, and Interleukin-6 (IL-6) Elisa Kit were purchased from BD Pharmingen (San Diego, CA, USA). Other reagents were of analytical or chromatography grade.

### 3.2. Construction of Plasmid

**Figure 1:**
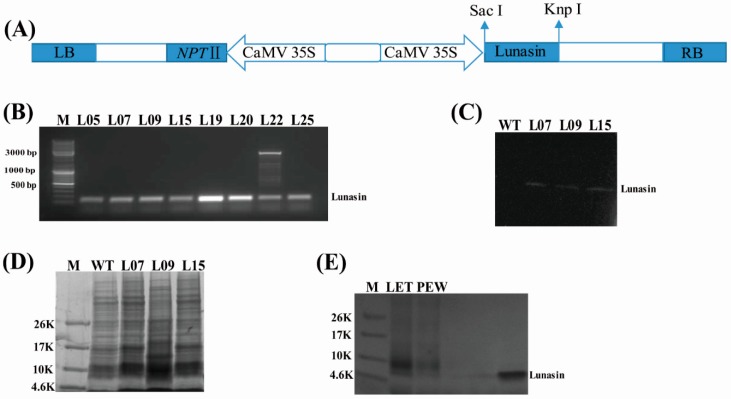
Construction of plasmid and molecular analysis of trans-lunasin rice. (**A**) T-DNA region of the plasmid PCAMBIA2301 for transformation. Lunasin was controlled by the CaMV35S promoter. RB: T-DNA right border. LB: T-DNA left border. (**B**) PCR demonstration of different transgenic rice lines. (**C**) Western blot analysis in the trans-lunasinrice lines. (**D**) Total protein was separated in a 12% polyacrylamide gel. (**E**) 4–20% SDS-PAGE of lunasin-enriched fractionfrom trans-lunasin rice (LET) and PEW (peptide-enriched fraction from wild type rice).

Total RNA of soybean was extracted using a Plant RNA Kit (Yuanpinghao Biotech, Beijing, China), then reverse transcribed into cDNA using a TransScript One-Step gDNA Removal and cDNA Synthesis SuperMix kit (TransGen Biotech, Beijing, China) according to the manufacturer’s instructions. The cDNAs were then used as templates to amplify the lunasincDNA sequence containing the SacI and KnpI digestion sites. Forward primer SacIun-F: CGAGCTCATGTCCAAATGGCAGCACCAGC and reverse primer KnpIun-R: GGGGTACCTCAGTCGTCGTCATCATCATC were used in the polymerase chain reaction. The PCR was conducted as follows: 95 °C, 5 min; 95 °C, 30 s; 58 °C, 30 s; 72 °C, 30 s, 30 cycles; 72 °C, 10 min. The PCR fragment product was cloned into vector Pcambia2301 double-digested with SacI and KnpI restriction enzymes under the control of the cauliflower mosaic virus 35S promoter (Figure 1A). Then, the plasmid Pcambia2301-lunasinwas transferred into *Agrobacterium tumefaciens* strain LBA4404 to transform rice according to a modified Agrobacterium-mediated protocol [27]. The variety ZH11 was used as an acceptor for the transformation.

### 3.3. Generation and Selection of Transgenic Lines Expressing Lunasin

Plants were determined to be transgenic using kanamycin resistance screening and PCR analysis. The PCR primers were designed based on the sequence of the lunasin gene by DNAMAN software. PCR was conducted as follows: 95 °C, 5 min; 95 °C 30 s; 58 °C, 30 s; 72 °C, 30 s, 30 cycles; 72 °C, 10 min, the predicated PCR product was 135 bp. Genomic DNA was isolated from transgenic rice lines and control rice ZH11 using acetyl trimethyl ammonium bromide (CTAB) extraction buffer [28]. The transgenic rice and control rice ZH11 were grown in a greenhouse, and we chose at least 20 T1 grains from each line to analyze the expression of lunasin by Western blot. In addition, the corresponding trans-lunasin rice embryon was germinated to generate T1 transgenic seedlings, which were cultured for generating T2 grains.

### 3.4. Protein Isolation and Western Blot

The T1 grains were pulverized using an ice-cold mortar and pestle, and then dissolved in PBS buffer through shaking it for 48 h at 4 °C. The resulting homogenate was centrifuged at 12,000× *g* for 15 min at 4 °C and then the supernatant was collected and freeze-dried for further analysis [14].

For Western blot analysis, the freeze-dried protein powder was dissolved in ultrapure water, separated in a 12% polyacrylamide gel, and stained in 0.1% Coomassie Brilliant Blue R-250 before transfer to a nitrocellulose membrane. The membrane was stringently washed with 20 mL PBS for 10 min in the shaking table (35 r/min), then the blot was blocked in PBS for 2 h with 5% nonfat dry milk. Primary rabbit polyclonal antibody lunasin epitope—EKHIMEKIQGRGDDDDD—was used as a hybridization probe. Goat anti-rabbit IgG-HRP was chosen as the secondary antibody. After hybridization and stringent washing with 20 mL PBS four times (each time period was 10 min), the blots were immerged in DAB solution to detect the target protein [29].

### 3.5. UPLC-MS/MS Analysis

The concentrations of the lunasin extract samples from the T1 trans-lunasin rice were detected by UPLC-MS/MS analysis. The lunasin standard was dissolved in ultrapure water by a series of gradient of concentrations (0–1000 pg·mL^−1^) and was used as the reference compound. UPLC coupled to a XEVO TQ-S mass spectrometer (Waters; Etten-Leur, The Netherlands) contained an electrospray ionization source (ESI). The ESI system employed a 4.0 kV spray voltage and a heated capillary temperature of 500 °C. Nitrogen was used as the nebulizing gas where the flow was 800 L/h. The MS instrument was operated in ion electrospray ionization mode with multiple reaction monitoring (MRM). The lunasin ion at *m*/*z* 1257.31, corresponding to the [M + 4H]^4+^ multicharged form, could be detected through MRM, used from 1257.3936 (parent, *m*/*z*) to 230.9777 (daughter, *m*/*z*). Data-dependent MS analysis was performed with normalized collision energy of 35%. The chromatographic analysis was carried out by using an UPLC peptide CSH C18 column (1.7 μm, 2.1 mm × 150 mm) whose mobile phase was a mixture of 0.1% trifluoroacetic acid in water (A) and 0.1% trifluoroacetic acid in acetonitrile (B). The flow rate was 0.1 mL · min^−1^ conducted in the following steps: 0 min 80% A, 2 min 40% A, 3.5 min 20% A, 5.5 min 20% A, and 7 min 80% A, until the initial condition was reached. Quantitative analysis of lunasin in the transgenic rice was detected by the measurement of the peak area. The calibration curve was available within a certain concentration range.

### 3.6. Protein Purification

Protein purification was conducted based on the method of Ren with minor modification [10]. The isolated lunasin supernatant from the T1 trans-lunasin rice was ultrafiltered with a Millipore ultrafiltration tube at 4 °C, which had the cut-offs of 1 kDa and 10 kDa. Then, the supernatant was collected and freeze-dried. Finally, the low-molecular-weight lunasin-enriched fraction from trans-lunasin rice (LET) could be obtained and its antioxidant activity and anti-inflammatory activity were assessed. The isolated peptides supernatant from the wild type rice was also ultrafiltered in similar ways. Then, the similar-molecular-weight peptide-enriched fraction from wild type rice (PEW) could also be obtained.

### 3.7. Protein Content Detection

After purification, the LET and PEW were dissolved in ultrapure water, respectively, then were separated in 4–20% precast gels to detect the variety and difference of the peptides in protein powder. The protein content of LET and PEW was detected using the BCA Protein Assay Kit according to the manufacturer’s instructions.

### 3.8. Flavone, Total Polyphenol, and Phenolic Acid Analysis

To clarify if there were some changes of functional components between WT and trans-lunasin, which contributed to the different antioxidant and anti-inflammatory activity, flavone, total polyphenol, and phenolic acid were measured. For the extraction, defatted powder of WT or trans-lunasin rice was dissolved in 50% ethanol, oscillated for 2.5 h at 80 °C, then centrifuged (5000 r/min) for 10 min. The supernatant was collected for further analysis. Total flavone content analysis was based on the method of Wu with some modification [30]. After the final reaction, absorbance was measured at 510 nm with rutin as the standard. We also detected the flavone composition in wild type or trans-lunasin rice through HPLC using an Apollo C18 column (5 μm, 250 mm × 4.6 mm) whose mobile phase was a mixture of 0.1% acetic acid in acetonitrile (A) and 0.1% acetic acid in water (B). The flow rate was 1.0 mL · min^−1^ and conducted as the following steps: 0 min 94% B, 10 min 90% B, 50 min 5% B, 65 min 94% B, and a 66 min strop step.

Total polyphenol content was determined using the Folin–Ciocalten method [31]. The extracted supernatant was mixed with Folin–Ciocalten reagent and sodium carbonate. Then, the reaction product was diluted with distilled water, and its absorbance at 725 nm was measured colorimetrically using gallic acid as a standard.The standard curve of gallic acid was drawn with the absorbance value as the *X* axis and concentration (ug/mL) as the *Y* axis.

In addition, the free and bound phenolic acid was also extracted based on the method of Kumar and Nayaka with some modifications [32]. Phenolic acid content and composition were measured through HPLC-UV using an Apollo C18 column (5 μm, 250 mm × 4.6 mm) whose mobile phase was a mixture of 0.05% trifluoroacetic acid in water (A) and 0.05% trifluoroacetic acid, 30% acetonitrile, 10% methyl alcohol in water (B). The flow rate was 1.0 mL · min^−1^ with the following steps: 0 min 10% B; 16 min 12% B; 25 min 38% B; 35 min 70% B; 40 min 86% B; 50 min 10% B, 60 min, and strop step. Flavone, polyphenol, and phenolic acid standards were dissolved in ultrapure water by a series of concentration gradients and were used as reference compounds. Quantitative analysis of flavone, polyphenol, and phenolic acid in the rice were detected by the measurement of the peak area. The calibration curves were available within a certain concentration range.

### 3.9. Antioxidant Activity Assay

The antioxidant activity was evaluated through detecting the DPPH radical scavenging ability, ABTS^+^ radical scavenging ability, and oxygen radical absorbance capacity. These tests are common methods to evaluate in vitro antioxidant activity [33,34,35,36]. For the DPPH assay, DPPH was dissolved in methanol, and LET and PEW were dissolved in ultrapure water to the final concentration gradients of 2, 4, 6, 8, 10, and 12 g·L^−1^, then the DPPH solution was mixed with the extract solution. After standing at room temperature in the dark for 30 min, the absorbance of the reaction product at 517 nm was measured colorimetrically using ultrapure water as a blank [37]. For the ABTS^+^ assay, LET and PEW were dissolved in ultrapure water to final concentration gradients of 0.5, 1, 1.5, 2, 3, 4, and 5 g·L^−1^, then the ABTS^+^ solution was mixed with the extract solution, and left to stand at room temperature in the dark for 6 min. The absorbance of the resulting solution at 734 nm was read with 95% ethanol as the control [38]. Moreover, the ABTS^+^ radical has different antioxidant characters than DPPH. The ABTS assay is superior to the DPPH assay when the sample contains hydrophilic antioxidant compounds [33]. The DPPH and ABTS^+^ radical scavenging activity of the lunasin standard and reduced glutathione were measured as the standard. The lunasin standard was dissolved in ultrapure water to final concentration gradients of 0.2, 0.4, 0.6, 0.8, and 1.0 g·L^−1^ for the DPPH assay and 20, 40, 60, 80, and 100 mg·L^−1^ for the ABTS^+^ assay. The reduced glutathione was dissolved in ultrapure water to final concentration gradients of 0.5, 1, 2, 4, 6, and 8 g·L^−1^ for the DPPH assay and 10, 20, 40, 80, 100, and 120 mg·L^−1^ for the ABTS^+^ assay.

ORAC activity was determined using the method described by Yao and Yang [39], where the LET and PEW were dissolved in 50% methanol to final concentrations of 0.5, 1, 1.5, 2, 3, and 4 g·L^−1^, then mixed with fluorescein solution and AAPH. The fluorescent value was read through a Synergy microplate fluorescence reader (Bio-Tek Instruments Inc., Winooski, VT, USA) with methanol as a blank. Trolox was used as the standard to detect the oxygen radical absorbance capacity of the samples. ORAC values were the mean concentration (μmol·L^−1^) of Trolox equivalents (TE) per gram of the samples.

### 3.10. Anti-Inflammatory Activities Assay

RAW264.7 macrophage cells were obtained from the Institute for Biological Science, Chinese Academy of Sciences (Shanghai, China). Before the anti-inflammatory activity assay, the RAW264.7 cells were cultured in PRMI1640 medium for the reproduction, which contained 10% FBS, 1% streptomycin, and 1% penicillin at 37 °C in 5% CO_2_. After reaching 70–80% confluence, the cells were seeded in a 96-well plate for 14 h. Then, the culture medium was removed and replaced with fresh medium containing the samples (LET, PEW, lunasin standard, and GSH) at different concentrations. After 2 h, the cells were treated with LPS (20 ug·mL^−1^). The nitric oxide concentration was measured in the culture supernatant after 24 h of co-incubation using the Griess reagent [40]. The quantification of nitric oxide was based on the standard curve of NaNO_2_ at 0–300 μM concentrations. The levels of MCP1 (CCL2), tumor necrosis factor-α (TNF-α), and interleukin-6 (IL-6) in the cell culture supernatants were measured using the ELISA kits. Every relevant monoclonal antibody against IL-6, MCP1 (CCL2), and TNF-α were detected based on the protocol of the ELISA kits. Absorbance was read at 450 nm. The standard curve of each cytokine was conducted in parallel with the samples.

### 3.11. Statistical Analysis

All of the above assays were repeated at least three times. Values were expressed as the means of three independent experiments ± SD. One-way analysis of variance (ANOVA), followed by Tukey HSD tests was employed to assess significant differences (* *p* < 0.05 and ** *p* < 0.01) using SPSS software (Statistics for Social Science) version 17.0. The statistical analyses of all graphical representations were performed using SPSS including the *p* value related to ANOVA, along with the *p* values related to the post hoc test.

## 4. Results and Discussion

### 4.1. Presence of Lunasin in Transgenic Rice

Through the genetic transformation, 56 independent transgenic rice lines were obtained after kanamycin selection and PCR detection. The PCR product was 138 bp, which was consistent with our prediction (Figure 1B). Due to the smaller numbers of some of the transgenic lines, we chose three independent lines (L07, L09, and L15) for further study as the three lines had enough seeds for further study. PCR and Western blot analysis showed that the lunasin gene was integrated into the rice genome and well expressed in transgenic rice lines L07, L09, and L15 (Figure 1C). We isolated the total protein from the trans-lunasin and wild type rice, then the total protein was separated in a 12% polyacrylamide gel, where the results showed that there was a variety of peptides in the protein extracts (Figure 1D). The purified protein (LET, PEW) and lunasin standard were separated in 4–20% SDS-PAGE, and it was shown that there may be two peptides in the purified sample, a peptide showing the same molecular weight as the lunasin standard in LET (Figure 1E). Additionally, other trans-lunasin lines continued to be sown for the seed reproduction.

Lunasin is a peptide isolated from soybean and has been found to also exist in other plants [11,12,13,14]. Given its bioactivities of anticancer, antioxidant, and anti-inflammatory, many studies have focused on its extraction from various plants or the chemical synthesis of lunasin. With the development of genetic engineering technologies, plant transformation has played an important role in developing new cultivars for our needs. In this study, we used the rice expression system to express the lunasin peptide and characterize its biological activity. The variety ZH11 was used as the acceptable material for the rice transformation. Then, we generated trans-lunasin rice using the Agrobacterium-mediated method. The results of the Western blot showed that the lunasin peptide was successfully expressed in trans-lunasin rice. Therefore, it is feasible to express exogenous lunasin using the rice expression system.

### 4.2. Lunasin Content Detection in Transgenic Rice Extract

**Figure 2:**
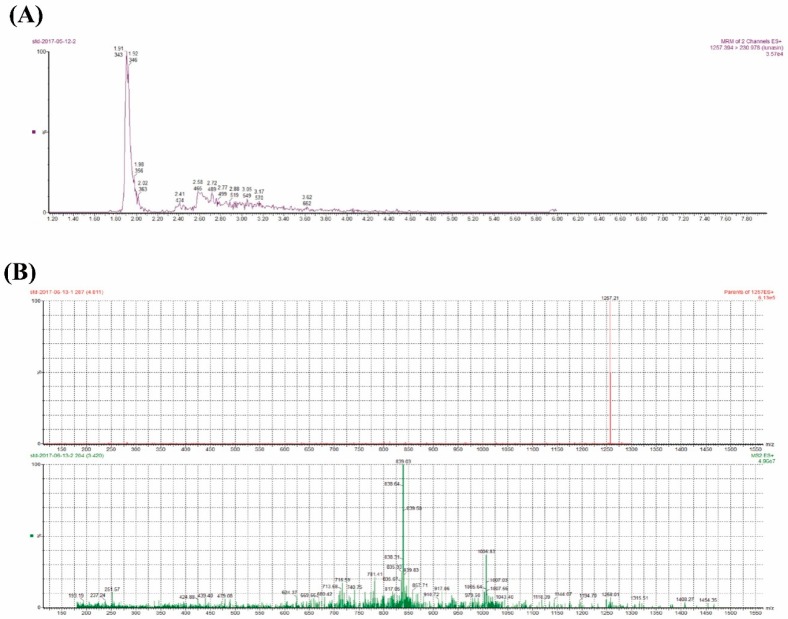
UPLC-MS/MS analysis. (**A**) Multiple reaction monitoring (MRM) chromatogram of the lunasin standard. (**B**) Mass spectrum acquired from the peak at 1.91 min in the MRM chromatogram of the lunasin standard.

**Figure 3:**
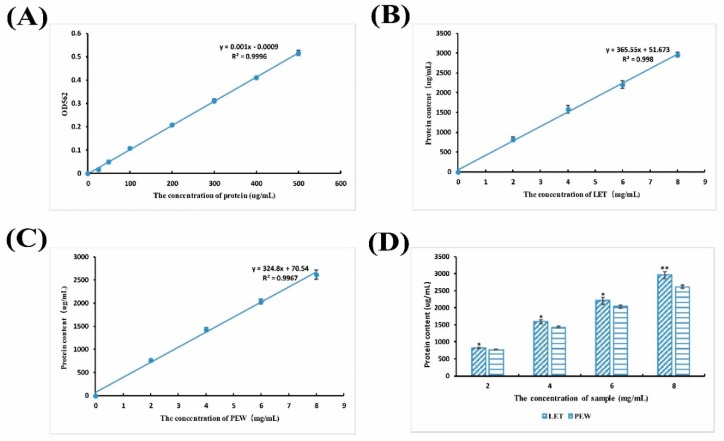
(**A**) Albumin standard curve in the BCA Protein Assay. (**B**) Protein content in LET. (**C**) Protein content in PEW. (**D**) Comparative analysis of the protein content in LET and PEW. Data are shown as the means of three independent experiments, the bars indicate ±SD. * *p* < 0.05 and ** *p* < 0.01 show significant differences between LET and PEW.

In the present study, the lunasin content in transgenic rice was detected by UPLC-MS/MS. AMRM chromatogram of the lunasin standard on the ESI generated [M + 4H]^4+^ at 1257.39 *m*/*z* and exhibited a peak at the retention time of 1.91 min (Figure 2A). The ESI mass spectrum related to this peak exhibited a multicharged profile (Figure 2B), which was consistent with the study previously reported in [41]. The MRM chromatogram of the lunasin extract from the transgenic rice grains appeared to exhibit a complicated signal because of the complex matrix in the extract, but a peak at the retention time of 1.78 mincould be found (Figure S4). We measured the lunasin content in the protein extracts from trans-lunasin rice, where it was 1.01 × 10^−3^ g·kg^−1^ dry rice flour with grease removal in the lunasin extracts. In addition, the protein content of LET and PEW was detected using the BCA Protein Assay Kit. As can be shown in Figure 3B,C, the protein content exhibited dose-dependency and significantly increased with the enhanced sample concentrations. Moreover, compared to PEW, LET showed a higher protein content (Figure 3D).

### 4.3. Flavone, Total Polyphenol, and Phenolic Acid Analysis

**Figure 4:**
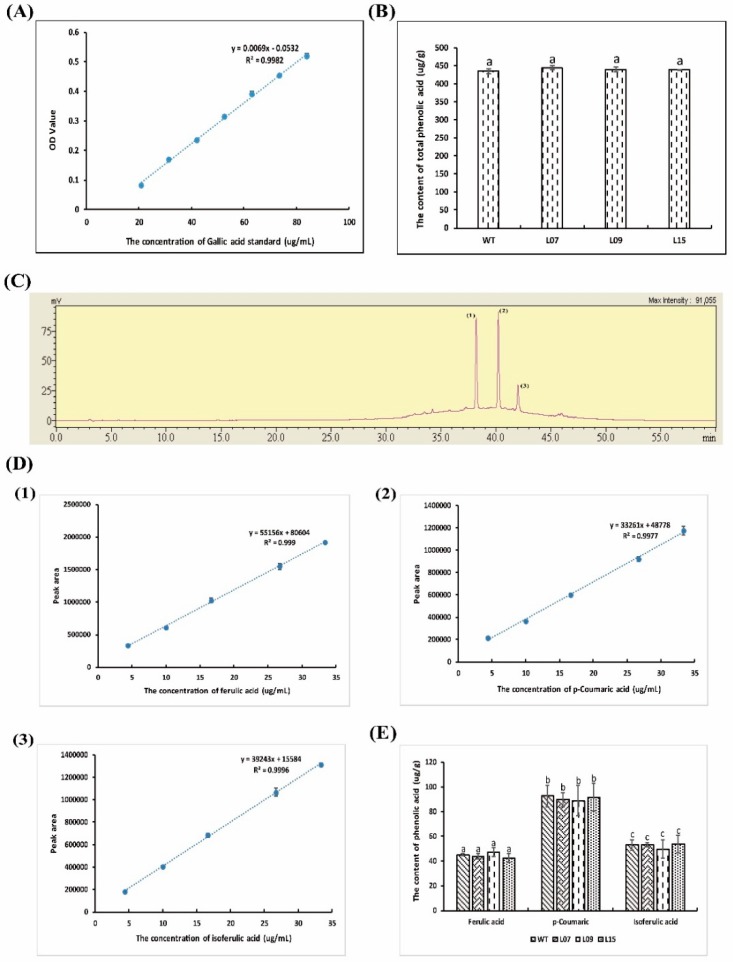
Phenolic acid analysis of the wild type and trans-lunasin rice. (**A**) Gallic acid standard curve in the total phenolic acid content assay. (**B**) Total phenolic acid content in the wild type and trans-lunasin rice. (**C**) Chromatographic analysis of the phenolic acid composition. (**1**) Ferulic acid; (**2**) *p*-Coumaric acid; (**3**) Isoferulic acid. (**D**) The standard curve of phenolic acids. (**1**) Ferulic acid; (**2**) *p*-Coumaric acid; (**3**) Isoferulic acid. (**E**) The composition and content of phenolic acids in the wild type and trans-lunasin rice. Data are shown as the means of three independent experiments, the bars indicate ±SD. The same letters above the columns between the different rice lines show no significant differences (*p* < 0.05).

Studies have shown that some flavones, polyphenols, and phenolic acids have high antioxidant and anti-inflammatory activity [42,43]. In this study, the content and composition of flavones, polyphenols, and phenolic acid were measured. The study showed that flavonoids were not detected either in the wild type or in the trans-lunasin rice, which may be due to the low flavonoids in rice (Figure S3). Total polyphenolcontent was detected with gallic acid as a standard (Figure 4A), where it was shown that there was no significant difference between the wild type and trans-lunasin rice (Figure 4B). Additionally, the content and composition of phenolic acids werealso measured. Three kinds of phenolic acid were detected in the rice including ferulic acid, *p*-coumaric acids, and isoferulic acid (Figure 4C). According to the standard curve of the phenolic acids (Figure 4D), the composition and content of the phenolic acids did not differ between the wild type and trans-lunasin rice (Figure 4E). The results demonstrated that there was no significant difference in the flavones and phenolic acids between the wild type and trans-lunasin rice. Lunasin content was the main difference between the wild type and trans-lunasin rice, which contributed to the different antioxidant and anti-inflammatory activity.

### 4.4. Antioxidant Activity Assay

**Figure 5:**
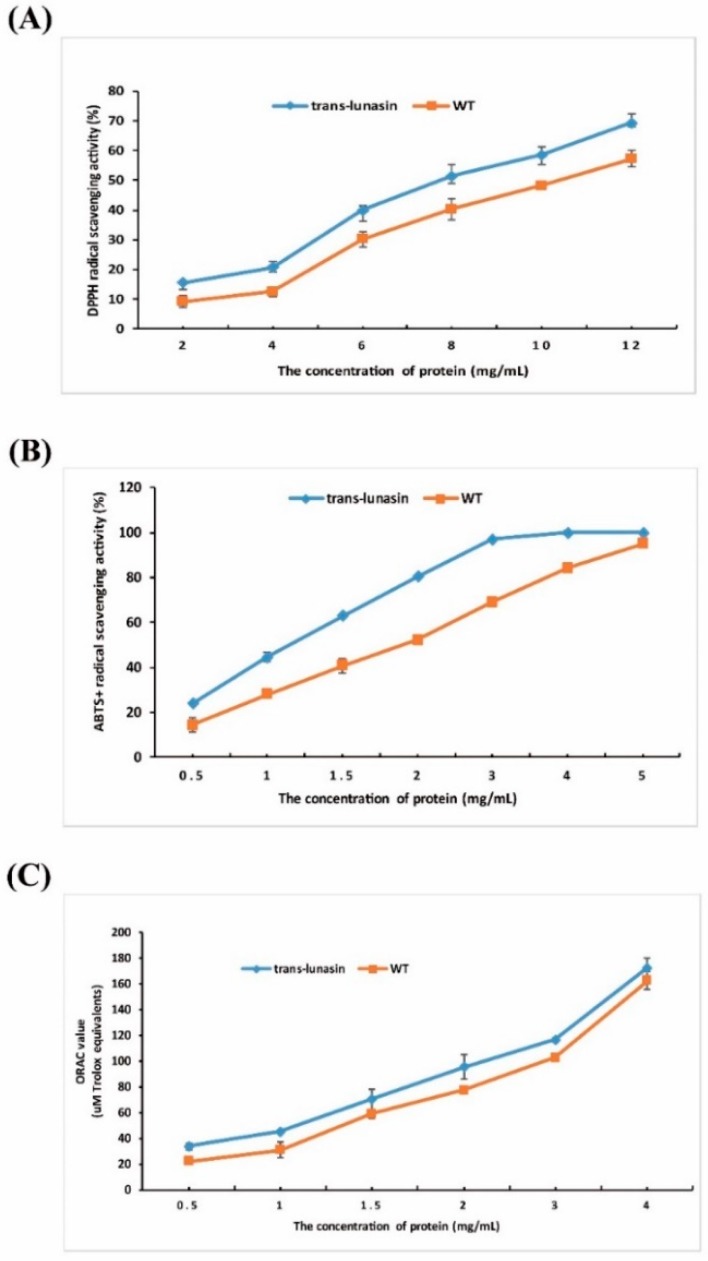
Antioxidant activity analysis of rice peptide extracts. (**A**) 1,1-diphenyl-2-picrylhydrazyl radical (DPPH) radical assay. (**B**) ABTS^+^ radical assay. (**C**) ORAC assay. Data are shown as the means of three independent experiments, the bars indicate ±SD.

In this study, we detected the antioxidant activity of LET and PEW. The antioxidant activity was evaluated comprehensively using three methods including the DPPH radical, ABTS^+^ radical, and ORAC assay. The results demonstrated that the radical scavenging activity was dose-dependent; with the increased concentration of the samples, the radical scavenging activity also increased. Moreover, because of the presence of lunasin, LET showed higher radical scavenging activity than PEW. In the DPPH radical assay (Figure 5A), LET exhibited a significantly higher DPPH radical scavenging activity (IC_50_, 8 g·L^−1^) than PEW (IC_50_, 11 g·L^−1^). GSH and the lunasin standard exhibited DPPH radical scavenging activity with an IC_50_ value of 1.96 g·L^−1^ and 0.51 g·L^−1^, respectively (Figure S1). In the ABTS^+^ radical assay (Figure 5B), LET exhibited ABTS^+^ radical scavenging activity with an IC_50_ value of 1.18 g·L^−1^, which was stronger than PEW with an IC_50_ value of 2 g·L^−1^. Both GSH and the lunasin standard exhibited high ABTS^+^ radical scavenging activity with IC_50_ values of 22.8 mg·L^−1^ and 0.5 g·L^−1^, respectively (Figure S1). Each protein has its own unique structure and amino acid sequence that determines its particular function. Therefore, due to the special structure and amino acid sequence, GSH and lunasin presented high antioxidant activity. Moreover, the GSH and lunasin standard were acquired through chemical synthesis, which has a high purity. In the ORAC assay, the ORAC values of LET were significantly higher than those of PEW (Figure 5C). The value was 170 μmol·L^−1^ Trolox equivalents when the concentration of LET reached 4 g·L^−1^. In a word, all the results suggested that the peptide extracts from the trans-lunasin rice (containing the lunasin peptide) showed stronger radical scavenging activities than those from the wild type rice. There was a good correlation between the DPPH, ABTS, and ORAC assays of the trials. However, because of the low purity, LET showed weaker antioxidant activity when compared to GSH and the lunasin standard.

The above results coincided with the previous studies. It has been shown that the lunasin peptide, isolated from various cereal plants, all exhibited antioxidant activity [14,18,44]. Soybean lunasin has anticancer, anti-oxidation, and anti-inflammatory activity [18,45]. Lunasin purified from oats showed similar antioxidant effects to the synthetic lunasin, which presented a high DPPH radical scavenging activity [14]. The lunasin peptide purified from *Solanum nigrum* L. can protect DNA from oxidative damage induced by Fe^2+^ ions and hydroxyl radicals. It can chelate Fe^2+^, and block the fenton reaction between Fe^2+^ and H_2_O_2_ [44].

### 4.5. Anti-Inflammatory Activity Assay

It has also been demonstrated that the lunasin peptide presents anti-inflammatory activity [7]. In this study, the anti-inflammatory activity of LET and PEW was evaluated by the LPS-stimulated immune reaction. LPS, an endotoxin existent in the outer membrane of Gram-negative bacteria, can stimulate phagocytic cells to produce signal mediators including NO and pro-inflammatory cytokines such as MCP1 (CCL2), TNF-α, and IL-6 [46]. NO is produced in large quantities mainly because of the nitric oxide synthetase, synthesized via the NF-κB regulation pathway [47]. Studies have shown that these signal mediators can take part in inflammatory reactions and play important roles in the pro-inflammatory reaction. Dia et al. found that lunasin isolated from defatted soybean flour had anti-inflammatory activity in RAW 264.7, released pro-inflammatory cytokines (TNF-α, IL-6, and MCP1), and NO production could be inhibited by the lunasin peptide [18]. The study of De Mejia and Dia showed that the LPS-stimulated inflammation reaction in RAW 264.7 macrophages could be inhibited via suppressing the NF-κB pathway by the lunasin peptide, purified from defatted soybean flour [6].

**Figure 6:**
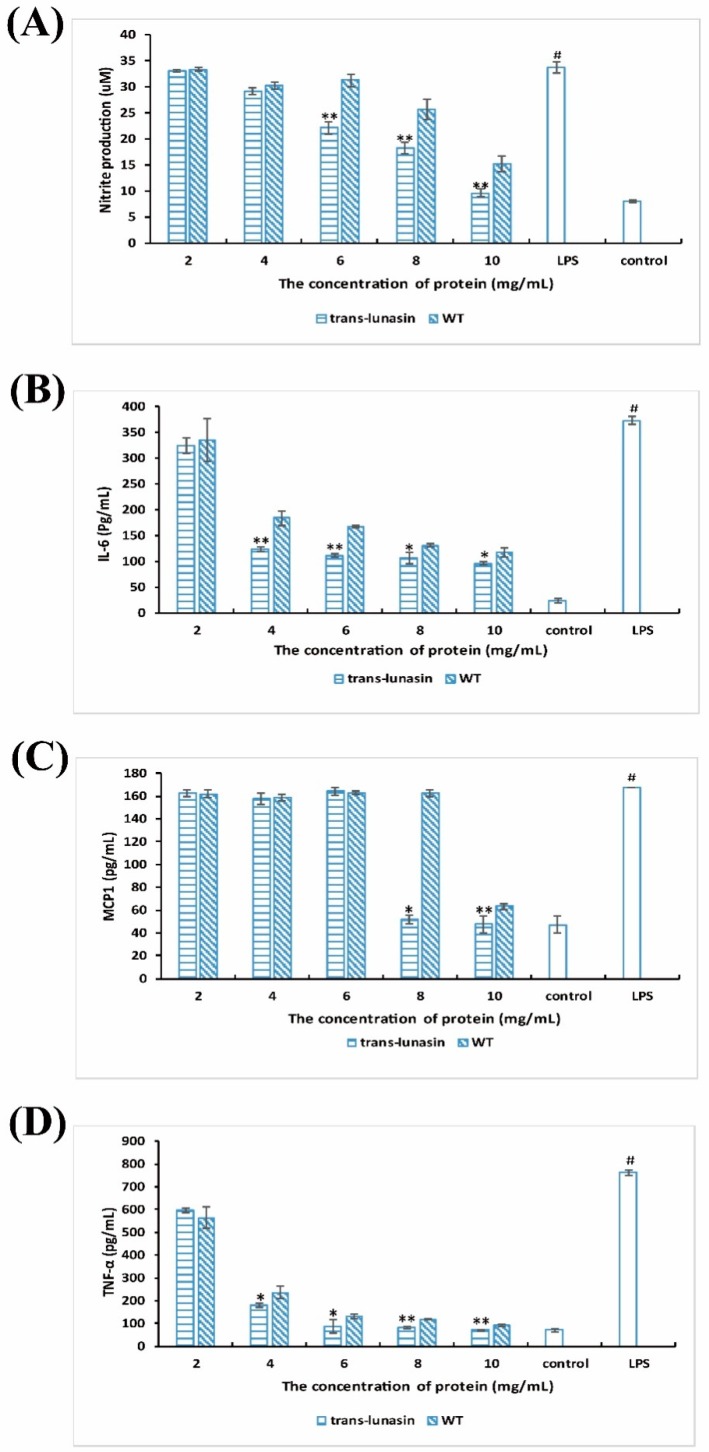
Anti-inflammatory activity analysis of the rice peptide extracts. The production of NO (**A**) and the release of pro-inflammatory cytokines including IL-6 (**B**), MCP1 (**C**), and TNF-α (**D**) in RAW264.7 cells were inhibited by the rice peptide extracts. Data are shown as the means of three independent experiments, the bars indicate ±SD. * *p* < 0.05 and ** *p*< 0.01 shows significant differences between the trans-lunasin rice and wild type rice, ^#^ *p* < 0.01 shows significant differences between the LPS-alone treated group and the vehicle control group.

As shown in Figure 6A–D, after the stimulation of LPS, the levels of NO, MCP1, TNF-α, and IL-6 were upregulated, and the upregulation was significantly inhibited by the rice peptide extracts. Moreover, LET exhibited a significantly higher inhibition rate. In the NO assay, LET exhibited an inhibition rate of 34.3% and PEW exhibited only 7.25% at the concentration of 6 g·L^−1^ (Figure 6A). In the IL-6 assays, the upregulation of IL-6 was significantly inhibited by LET up to 66.8% at the concentration of 4 g·L^−1^, but the inhibition rate of IL-6 by PEW was 50.6% at the same concentration (Figure 6B). In the MCP1 assay, LET exhibited an inhibition rate of 69.2% at the concentration of 8 g·L^−1^, but the PEW exhibited only 2.8%. However, the inhibitory effect of MCP1 by LET was not seen at the concentrations of 2 g·L^−1^, 4 g·L^−1^, and 6 g·L^−1^ (Figure 6C). In the TNF-α assay, the inhibition rate by LET was 76.2% at the concentration of 4 g·L^−1^, and the inhibition rate by PEW was 68.8% (Figure 6D).

In addition, the anti-inflammatory activities of GSH and the lunasin standard were also measured. We chose to detect the levels of NO, MCP1, and TNF-α after the co-culture of GSH/lunasin with RAW 264.7, where the upregulation of NO, MCP1, and TNF-α in the LPS-stimulated inflammation was significantly inhibited by GSH and the lunasin standard. In particular, when the lunasin standard reached 1 g·L^−1^, the inhibition rate of NO, IL-6, and MCP1 was 24.2%, 46.3%, and 55%, respectively (Figure S2). Furthermore, when GSH reached 4 g·L^−1^, the inhibition rate of NO, IL-6, and MCP1 was 19.7%, 55.5%, and 59.1%, respectively (Figure S2). Moreover, with the increase of the GSH/lunasin concentration, the inhibition rate also increased.

The results showed that LET showed a significantly higher inhibition rate of NO production and release of pro-inflammatory cytokines (TNF-α, IL-6, and MCP1) than PEW, which demonstrated that trans-lunasin rice peptide extracts exhibited a great anti-inflammatory activity on macrophage cells. However, when compared to the GSH and lunasin standard, LET showed lower anti-inflammatory activity, which resulted from the low purity of the lunasin peptide in LET. In addition, rice peptide extracts (both LET and PEW) showed antioxidant and anti-inflammatory activities, which may be due to the presence of other functional peptides, shown in 4–20% SDS-PAGE (Figure 1E). Experimental evidence on the functional substance in rice peptide extracts is lacking, so further studies should investigate the bioactive peptide in rice extracts in greater depth.

## 5. Conclusions

We inserted soybean lunasin into the rice genome and showed that it was feasible to use the rice expression system to express the exogenous lunasin in rice. The trans-lunasin rice peptides extracts showed enhanced antioxidant and anti-inflammatory activity, so the lunasin-overexpressing rice seems to be a candidate resource for application in functional food. Our results provide a new germplasm resource for the improvement of function in rice. Rice rich in lunasin is beneficial for human health, and could be used as a functional food in the diets of cancer and obese patients in the future.

## Supplementary Materials

The following are available online: Figures S1–S4.

## Abbreviations

LET	Lunasin-enriched fraction from trans-lunasin rice
PEW	Peptide-enriched fraction from wild type rice
PCR	Polymerase chain reaction
MCP1/CCL2	Monocyte chemoattractant protein chemokine (C-C motif) ligand 2
TNF-α	Tumor cell necrosis factor-α
IL-6	Interleukin-6
UPLC-MS/MS	Ultra Performance Liquid Chromatography with Tandem Mass Spectrometry
XEVO TQ-S	Triple Quadrupole
AAPH	2,2′-Azobis(2-methylpropionamidine)dihydrochloride
RPMI-1640	Roswell Park Memorial Institute 1640
FBS	Fetal Bovine Serum
UPLC	Ultra Performance Liquid Chromatography
