# Supplementary material for: Expression of Bioactive Lunasin Peptide in Transgenic Rice Grains for the Application in Functional Food
Source: Molecules. 2018 Sep 17;23(9):2373. doi: 10.3390/molecules23092373 (PMC6225134; doi:10.3390/molecules23092373)
Supplement: Supplementary file 1 [file molecules-23-02373-s001.pdf]

Expression of bioactive lunasin petide in transgenic rice grains for the application in functional food

Guixing Ren[1,2,*,†,], Yuqiong Hao[2,†], Yingying Zhu[2,3], Zhenxing Shi[2,4] and Zhaogang[1,5,*]

1 College of Pharmacy and Biological Engineering, Chengdu University, No.1 Shilling Road, Chenglo Avenue, Longquan District, Chengdu City, Sichuan Province, China, 610106.
2 Institute of Crop Science, Chinese Academy of Agricultural Sciences, No.80 South Xueyuan Road, Haidian, Beijing 100081, People's Republic of China.
3 Precision livestock and nutrition unit, Gembloux Agro-Bio Tech, TERRA Teaching and Research Centre, University of Liège, Passage des Déportés, 2. 5030 Gembloux, Belgium.
4 Laboratory of Biomass and Green Technologies, Gembloux Agro-Bio Tech, University of Liege, Passage des Déportés, 2. 5030 Gembloux, Belgium
5 Key Laboratory of Coarse Cereal Processing, Ministry of Agriculture, Chengdu University, No.1 Shilling Road, Chenglo Avenue, Longquan District, Chengdu City, Sichuan Province, China, 610106.
* Correspondence: renguixing@cdu.edu.cn (G.R.); zhaogang@cdu.edu.cn (Z.G.); Tel.: +86-10-6211-5596 (G.R.); Fax: +86-10-6215-6596 (G.R.)
† These authors have contributed equally to this work.

Supplementary Materials:

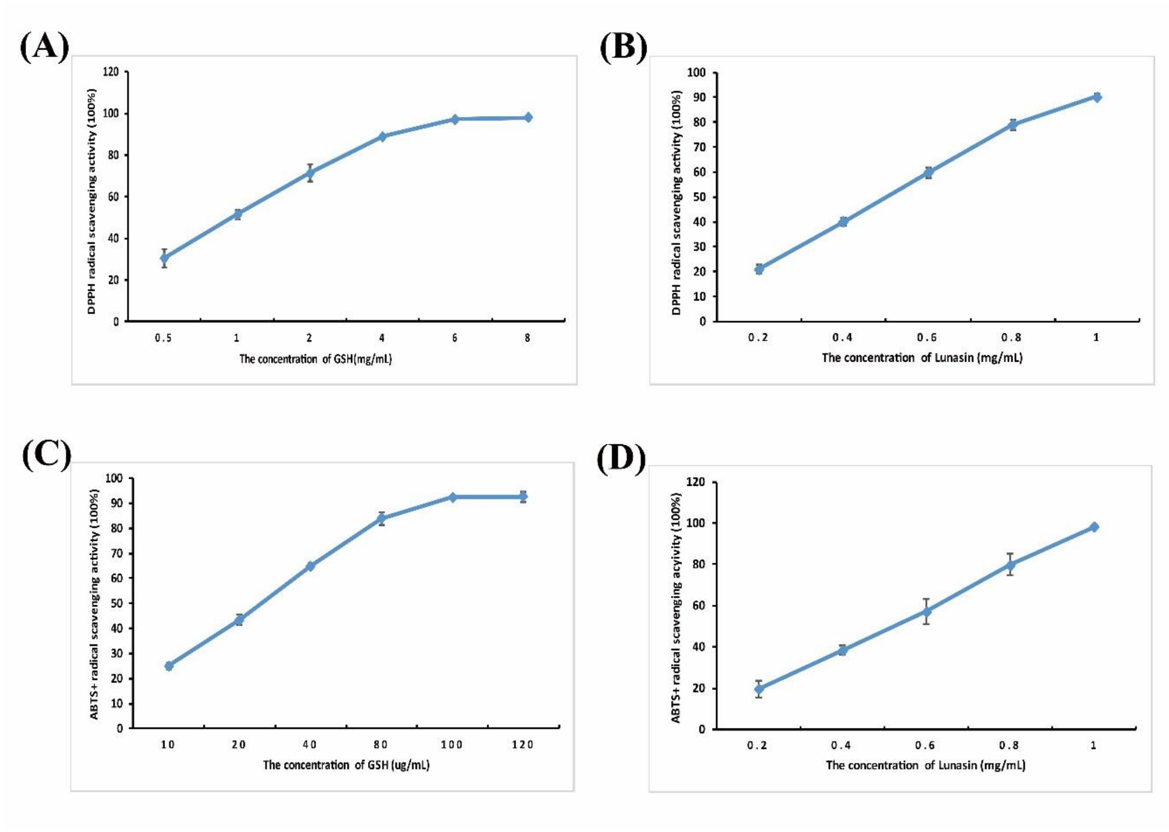

Figure S1. Antioxidant activity analysis of reduced glutathione (GSH) and lunasin standard. (A) DPPH radical assay of GSH. (B) DPPH radical assay of lunasin standard. (C) $ABTS^+$ radical assay of GSH. (D) $ABTS^+$ radical assay of lunasin standard.

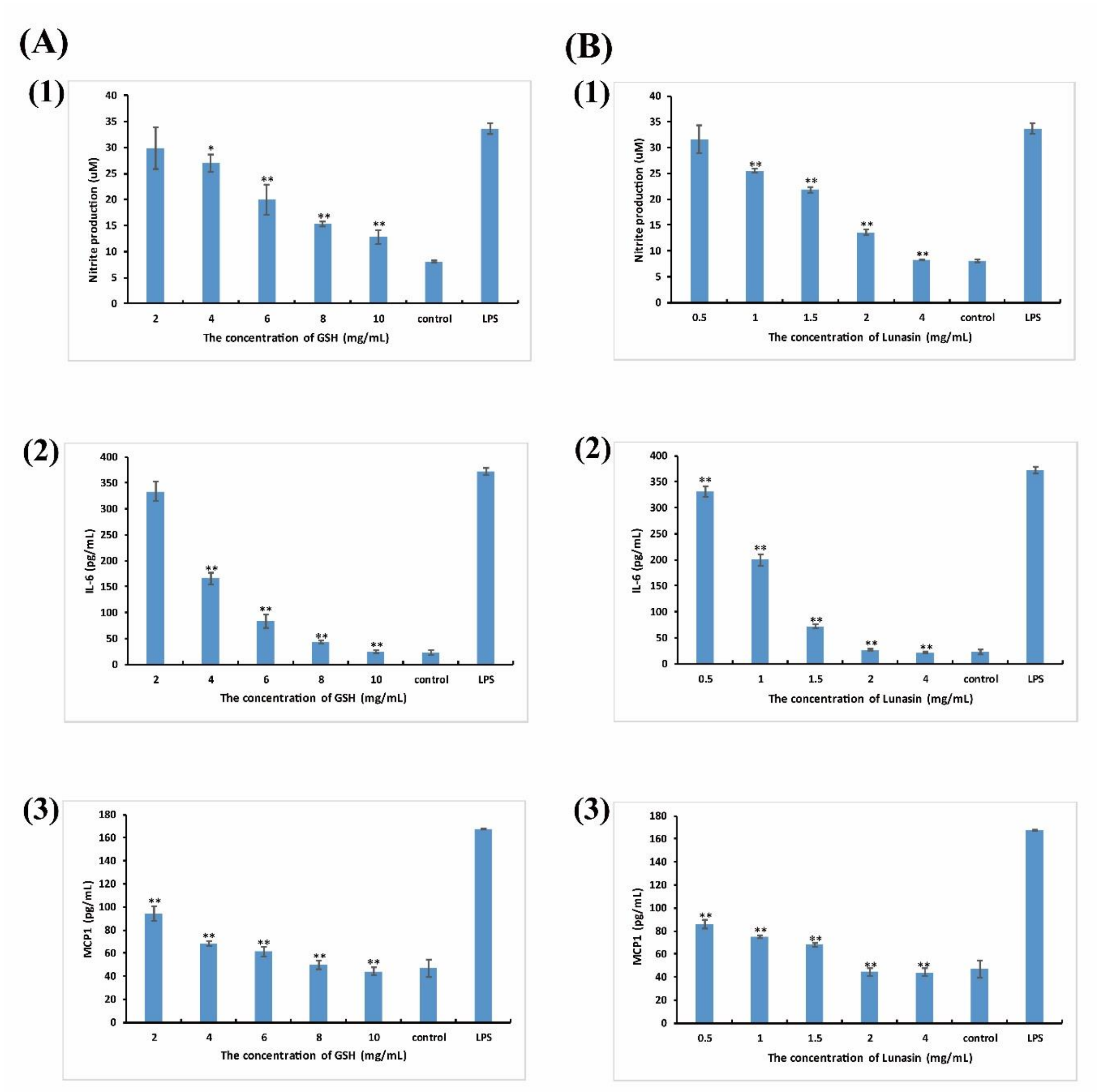

Figure S2. Anti-inflammatory activity analysis of reduced glutathione (GSH) and lunasin standard. (A) Production of NO (1) and the release of pro-inflammatory cytokines including IL-6 (2), MCP1 (3) in RAW264.7 cells were inhibited by GSH. (B) Production of NO (1) and the release of pro-inflammatory cytokines including IL-6 (2), MCP1 (3) in RAW264.7 cells were inhibited by lunasin standard. Data are shown as the means of three independent experiments, the bars indicate ±SD. *P < 0.05 and **P < 0.01 show significant differences between the GSH/ Lunasin standard and the LPS-alone treated group.

(A)

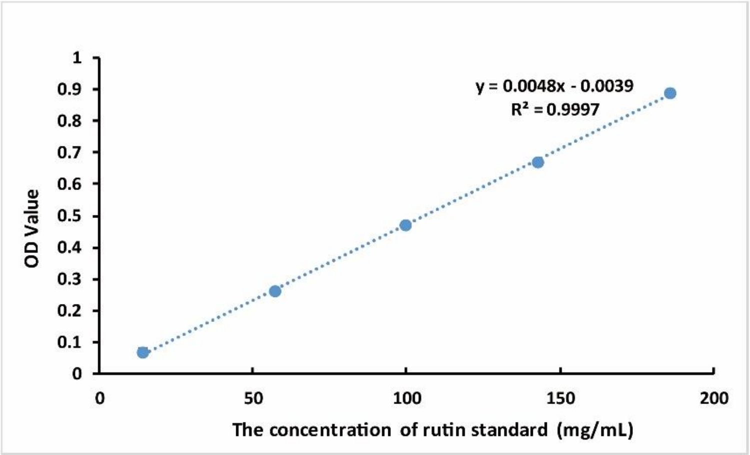

(B)

	WT	L07	L09	L15
OD510	0.0129833	0.0075833	0.0058833	0.0102833
	0.0147833	0.0100833	0.0079833	0.0122833
	0.0142833	0.0083833	0.0072833	0.0109833

Figure S3. Flavone analysis of wild type and trans-lunasin rice. (A) Rutin standard curve in total flavone content analysis. (B) OD value at 510nm in Flavone content assay.

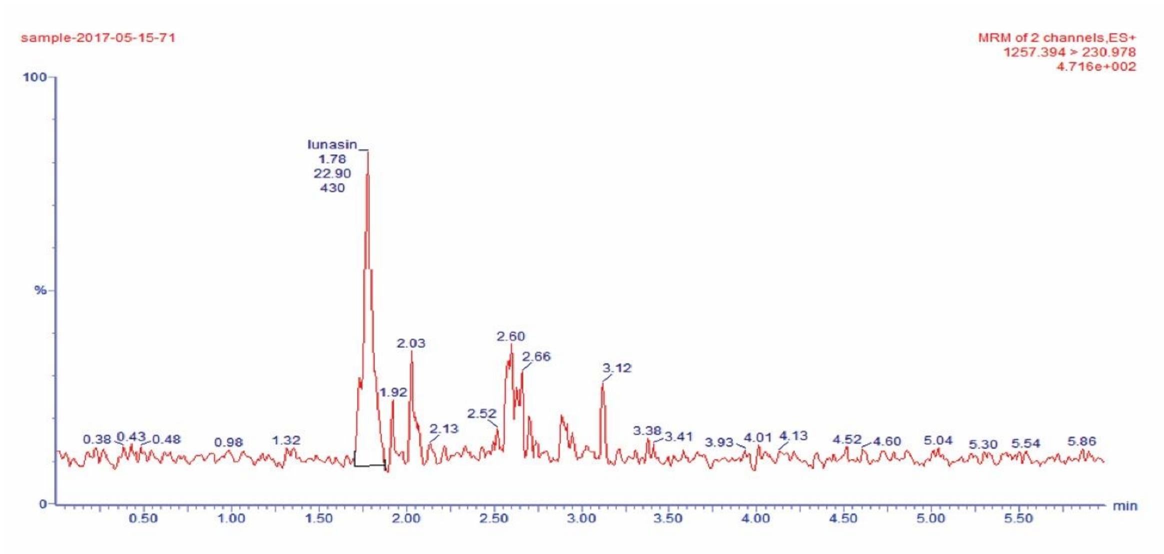

Figure S4. MRM chromatogram of trans-lunasin rice extract.
